# Expertise influences congruency monitoring during action observation at the motor level

**DOI:** 10.1093/scan/nsab078

**Published:** 2021-07-01

**Authors:** Qiwei Zhao, Yixuan Wang, Yifan Chen, Yingying Wang, Chenglin Zhou, Yingzhi Lu

**Affiliations:** School of Psychology, Shanghai University of Sport, Shanghai 200438, China; School of Psychology, Shanghai University of Sport, Shanghai 200438, China; School of Psychology, Shanghai University of Sport, Shanghai 200438, China; School of Psychology, Shanghai University of Sport, Shanghai 200438, China; School of Psychology, Shanghai University of Sport, Shanghai 200438, China; School of Psychology, Shanghai University of Sport, Shanghai 200438, China

**Keywords:** congruency monitoring, action understanding, sports expertise, N400, theta oscillation

## Abstract

Congruency monitoring of action occurs in individuals with relevant motor experience while observing actions. However, it remains unclear whether congruency monitoring can occur at the motor level and the extent to which expertise contributes. Here, we examined the behavioral performance and electrophysiological brain activity of individuals with and without domain-specific expertise when judging the action congruency of occluded video clips of a snowboard halfpipe trick and normal walking. For the halfpipe trick, experts exhibited better task performance and greater midline theta oscillations before possible incongruency compared with controls. Source reconstruction for the theta oscillation revealed a stronger activation in the middle and superior frontal gyrus for experts in response to incongruency compared with controls. Incongruent halfpipe actions elicited a higher N400 amplitude in experts compared with congruent actions, while no such differences were observed in controls. Source reconstruction revealed the activation in the board frontal regions and middle temporal gyrus for experts. These findings suggest that congruency monitoring can occur at the motor level during action observations and is modulated by individual expertise. The modulation of expertise reflects in the special N400 effect and midline theta oscillation.

## Introduction

In social interactions, it is fundamentally important to understand other people’s actions efficiently to plan our own actions. Individuals can use their experience to extract the goals and intentions of others by viewing their actions and predict the probable outcome of actions (Hauser and Wood, 2010). For example, in a rapidly changing table tennis game, players must predict the ball trajectory through the opponent’s serve and then organize their response actions (Zhao *et al.*, 2018). However, the ball trajectory may deviate from the initial prediction. Consequently, a continuous congruency monitoring process during the action observation helps individuals to detect any disharmony and make timely adjustments to unexpected hits. This process reflects the comparison between the actual perceived scenario and the expectation based on an individual’s motor repertoire.

The congruency monitoring process might contribute to action understanding, which is influenced by dynamic changes in kinematic and contextual information. Grafton (2007) proposed that actions can be hierarchically organized into different levels, ranging from concrete to abstract. Kilner’s two-way model (2011) of action understanding states that concrete information is encoded in a dorsal pathway, while abstract information is encoded in a ventral pathway. Concrete information is represented by kinematic and motor levels, which include the trajectory and velocity profile of actions or related muscle activities. In contrast, abstract information is represented by the goal and intention levels, which include the meaning and goal for executing a given action. However, it remains unclear whether and how these levels were involved in congruency monitoring.

On the basis of existing studies, it is reasonable to speculate that congruency monitoring can occur at the goal level (i.e. the abstract meaning of a given action), including inferring the goal through concrete information and monitoring whether the goal is consistent with the expectation based on prior knowledge and contextual information. The mirror neuron system is a dedicated brain network considered to underlie action understanding (Rizzolatti and Sinigaglia, 2016). During action observation, this system registers subtle differences in movement kinematics and helps to identify the goal (Cavallo *et al.*, 2016; Koul *et al.*, 2018). In addition, when an unreasonable scene is presented after a continuous action (e.g. an unsuitable posture is performed or the wrong tool is manipulated for reaching the action goal), the incongruency between the actual perceptual scenario and the expected action goal might arise, eliciting related neural activity (Sitnikova *et al.*, 2008; Amoruso *et al.*, 2018). Consequently, in addition to the presented action, an object that reflects contextual meaning and implicit abstract information helps to identify the goal of an action. A previous study showed that, when viewing an action, brain activation with the goal overlaps directly with object-related activation, but not with movement-related activation (Nicholson *et al.*, 2017). However, if congruency monitoring does occur at the motor level (i.e. concrete information on actions), incongruency in actions that have no clear goal or object information should also be perceived. Previous studies showed that elite gymnasts judged the quality of rhythmic gymnastics movement with higher accuracy when compared with novices based on details of action kinematics (Babiloni *et al.*, 2009). Similarly, dancers could identify subtle incongruencies in dance movements (Amoruso *et al.*, 2014; Orlandi *et al.*, 2017). Thus, congruency monitoring likely also occurs at the motor level, with processing being influenced by an individual’s expertise.

In contrast to unfamiliar actions, the motor repertoire of an experienced individual facilitates understanding actions (Buccino *et al.*, 2004). Some studies have shown that the activation of the mirror neuron system is higher when experts view movements that they had been trained on compared with movements that they had not been trained on (Calvo-Merino *et al.*, 2005, 2006). During observation, incongruencies in actions can be detected by experienced individuals, eliciting action-N400 activity. For instance, incorrect basketball shot movements were detected by skilled basketball players (but not by novices) and led to a higher N400 amplitude over the anterior-frontal area of the brain (Proverbio *et al.*, 2012). Similarly, skilled dancers could identify subtle errors in dance movements more accurately and had higher N400 amplitudes compared with novices (Amoruso *et al.*, 2014). Therefore, N400 activity can be considered as an electrophysiological indicator of incongruency detection and modulated by expertise, reflecting the construction of action meaning from previous experiences and current contextual information (Amoruso *et al.*, 2013). The neural sources of action-N400 activity involve widely distributed semantic and motor regions (Proverbio *et al.*, 2010, 2012; van Elk *et al.*, 2010; Amoruso *et al.*, 2014; Orlandi *et al.*, 2017). In particular, the activation of the superior temporal gyrus and middle temporal gyrus is modulated by an individual’s expertise, reflecting action processing in the ventral pathway (Amoruso *et al.*, 2014). Consistent with this, a functional magnetic resonance imaging study detected a greater activation of the middle temporal gyrus, inferior parietal lobe and ventromedial prefrontal cortex when table tennis experts viewed the incongruent ball trajectory compared with the congruent trajectory (Wang *et al.*, 2019). In addition, the theta-band oscillation over the midfrontal cortex reflects conflict detection and resolution processes (Cohen and Donner, 2013) and is modulated by an individual’s expertise during action observation. Brain dynamics during action anticipation was examined by presenting participants with edited videos of table tennis serve movement in a previous study (Lu *et al.*, 2020). Table tennis experts judged the action congruency more accurately than controls and showed elevated midline theta oscillations sourced from broad frontal regions when observing the outcome of serves after the ball–racquet contact. This suggested the theta oscillation is sensitive to whether the outcome of action exceeds an individual’s expectation or not and reflects congruency monitoring during action observation.

In Lu’s study (2020), the goal of the table tennis opponent’s serve action (i.e. serving the ball to a specific position) could be inferred from kinematic information. Thus, it is not sufficient to explain the congruency monitoring at the motor level. Based on the above evidence, it can be seen that incongruency in actions without clear goals or intentions (e.g. dance movements) could be detected by experienced individuals, involving a matching between the viewed action and own motor repertoire (Amoruso *et al.*, 2014; Orlandi *et al.*, 2017). N400 and theta oscillation, as indicators of neural activity, are involved in the processing of action congruency. In sum, we hypothesize that (i) during action observation, congruency monitoring can occur at the motor level, with processing enhanced by expertise, and (ii) at the neural activity level, the modulation of expertise reflects in the special N400 and theta oscillation activities.

To examine our hypotheses, we investigated how the congruency monitoring process occurs at the motor level and how it is influenced by expertise. To avoid the ambiguity of the action level, expert snowboard halfpipe athletes and individuals with no snowboarding experience (controls) were recruited to observe non-goal-directed actions and to make judgments about the congruency of the viewed actions. The electrophysiological activity was recorded throughout the observations. We selected actions of snowboard halfpipe as stimuli because (i) a snowboard halfpipe trick has no clear goal, (ii) participants could view whole-body movements with no additional object information available during the observation, (iii) it is difficult for individuals with no specific motor repertoire for this activity to predict upcoming movements and (iv) snowboarding halfpipe competitions are more of a niche sport compared with other close-skilled sports such as dance and gymnastics; thus, controls were expected to have limited to negligible sensorimotor or visual experience with this sport. To identify the modulatory effects of expertise on congruency monitoring, we used walking movements as a control condition. We expected that (i) both experts and controls would identify incongruencies and show an increase in theta oscillations during the observation of walking actions, reflecting congruency monitoring at the motor level; (ii) experts would show a greater theta oscillation compared with controls when observing incongruent halfpipe actions, sourced from broad frontal regions; and (iii) the N400 effect would only be detected in the expert group when observing halfpipe actions, with the incongruency-related effect being sourced from semantic regions such as the middle temporal gyrus.

## Method

### Participants

Fifteen elite snowboarding halfpipe athletes aged 16–27 years (mean 20.07 ± 3.84 years; 9 women) from China’s national team were recruited as the expert group, and 15 college students aged 18–25 years (mean 19.47 ± 1.88 years; 8 women) were recruited as the control group. Experts had at least 6 years (mean 9.53 ± 3.90 years) of professional training experience and qualified as a National Player at the First Grade or above. Controls recruited from the Shanghai University of Sport had no training in snowboard halfpipe tricks or any other similar sport and had hardly ever watched relevant competitions. All participants except two were self-reported right-hand dominant. All participants possessed the normal or corrected-to-normal vision and reported no neurological or psychiatric history. Participants signed a written consent form before taking part in the study and received a payment of 100 RMB after completing the tasks. The privacy rights of all participants were respected. This experiment was approved by the Ethics Committee of the Shanghai University of Sport (Approval No. 102772019RT011), and the study was conducted in accordance with the Code of Ethics of the World Medical Association (Declaration of Helsinki).

### Stimuli

**Fig. 1.**
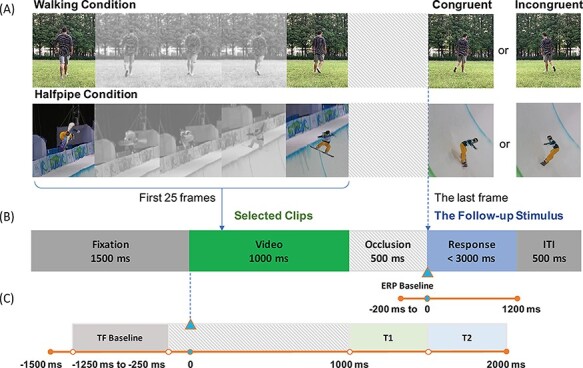
(A) Each clip consisted of the first 25 frames of the selected video, while the follow-up stimulus was the last frame of each clip (the 38th frame). (B) In this two-alternative forced-choice task, the presented video was temporarily occluded and then connected to a congruent or an incongruent follow-up stimulus. Each trial started with a participant fixating on a crosshair presented on a computer screen for 1500 ms. A video clip was then played for 1000 ms and was occluded for 500 ms. The follow-up stimulus was then presented, and the participant had to respond within 3000 ms of the onset of the stimulus. The intertrial interval (ITI) was 500 ms. (C) For the ERP analysis, the baseline was the period 200 ms before the onset of the follow-up stimulus. In the TF analysis, the onset of the video clip was considered zero, the baseline considered to extend from −1250 to −250 ms, pre-stimulus time window (T1) was the 500 ms duration of the occluded video clip and post-stimulus time window (T2) began at the onset of the follow-up stimulus and ended 500 ms later.

The stimuli comprised 1500 ms video clips displaying a snowboarding halfpipe trick or a typical walking motion (Figure 1). Ten halfpipe trick clips from the broadcast video of the Vancouver and Sochi Winter Olympic snowboarding halfpipe preliminary and final competitions were selected as halfpipe stimuli. The criteria for clip selection were as follows: (i) the clip presented one complete snowboard halfpipe trick; (ii) the clip was recorded from a single, unchanging camera angle; (iii) the movement in the clip was fluent and (iv) the athlete in the clip was not a participant or a teammate of any participant in the present study. In addition, two video clips of a male (25 years old) walking and turning with his natural gait were selected (each had a resolution of 1920 × 1080 pixels and was captured at 60 frames per second, fps). The male volunteer initially stood on an empty lawn but then walked away from the video recorder and turned to the left or the right. All the selected clips were edited using the following five steps with the video editor Wondershare Filmore 9 (Wondershare China, Guangdong, PR China): (i) the audio tracks were muted; (ii) the frame size and rate were changed to a video resolution of 640 × 360 pixels at 25 fps (40 ms per frame, with each clip consisting of 38 frames); (iii) the first 1000 ms of each clip (1–25 frames) was selected as the video stimulus, and the last frame in each clip was selected as the follow-up stimulus; (iv) each clip was occluded by deleting frames 26–37 from 1000 to 1500 ms and (v) the original follow-up stimulus (last frame in each clip) was designated the congruent stimulus, whereas a mirror image of the last frame was designated the incongruent stimulus (Figure 1A).

### Procedure

Participants were asked to judge the congruency between the action in the video clip and that in the final follow-up stimulus. Each trial started with a visual fixation crosshair presented in the center of the screen for 1500 ms. An action video was then presented for 1000 ms, followed by a blank screen for 500 ms, and finally, the follow-up stimulus (congruent or incongruent) was presented (Figure 1B). Participants were asked to respond within 3 s of the onset of the follow-up stimulus. They pressed the letter ‘F’ on a computer keyboard using their left index fingers when they judged that the follow-up stimulus was congruent with the action on the video and pressed the letter ‘J’ using their right fingers when the follow-up stimulus was judged to be incongruent with the action on the video. An intertrial interval of 500 ms followed immediately after the pressed key was released. The task contained two conditions, the halfpipe and the walking. Each condition contained two blocks, and each block included 40 trials with a 1:1 ratio of congruent to incongruent stimuli presented in random order. The order in which the conditions were presented was balanced among the participants.

After each condition was completed, participants were asked to fill out a questionnaire containing four questions with a visual analog scale (VAS) for the self-assessment of task performance and familiarity with the stimuli. Each participant filled out the questionnaire twice: once for the walking condition and once for the halfpipe trick. The VAS was a continuous scale composed of a horizontal line 100 mm in length, anchored by two verbal descriptors (‘not at all’ and ‘very’). Participants rated their subjective feelings on four questions. Question 1 (‘How familiar are you with the movements in the task?’) was used to assess their familiarity with the presented stimuli. Questions 2–4 were used to determine their subjective assessments of their task performance (Question 2: ‘How confident are you in your judgment?’, Question 3: ‘How much do you imagine yourself doing the same movement during the task?’ and Question 4: ‘How difficult do you think the task is?’).

### Data recording

During the experiment, participants sat in a dimly light room ∼70 cm from a 24-inch LCD monitor (resolution, 1920 × 1080 pixels; refresh frequency, 60 Hz). E-prime, version 3.0 (Psychology Software Tools, Inc., Sharpsburg, PA), was used to present all visual stimuli with the corresponding triggers and to record both response accuracy and reaction time via the keypress. The electroencephalogram (EEG) signals were recorded using Brain Vision Recorder, version 2.0 (Brain Products GmbH, Germany), at a sampling rate of 1000 Hz from 64 electrodes placed on the scalp according to the international 10-10 system. The EEG record was referenced online against the FCz site and was grounded at the AFz site. One electrode was placed on the left mastoid and another on the right mastoid for offline re-referencing. The vertical electrooculogram was recorded just below the left eye. The horizontal electrooculogram was recorded at the outer canthus of the right eye. Electrode impedances were kept below 5 kΩ for the duration of the experiment. The E‐Prime and the EEG system software timing were synchronized with the video stimulus onset.

### Data processing and statistical analysis

Statistical analysis was performed using SPSS, version 20.0 (IBM SPSS, Inc., Chicago, IL, USA). For all behavioral and EEG data analyses, *P* values less than 0.05 were considered statistically significant. The sphericity assumption was evaluated using Mauchly’s test, and the Greenhouse–Geisser correction for the degrees of freedom was used in cases of non-sphericity. The Bonferroni correction was used to correct for multiple post hoc comparisons. The effect size for the statistically significant factors was estimated using partial eta squared ($\rm{{\eta}_{p}^{2}}$).

#### Behavioral performance and questionnaire.

Accuracy, reaction time and the sensitivity index (*d*′) were used as indicators of behavioral performance. The averaged reaction time is considered –222 ms when participants perform a simple reaction task by pressing a key for any presented stimulus without any discrimination (Di Russo *et al.*, 2019). To ensure that participants exhibit a valid discrimination process in this two-alternative forced-choice task (2AFC) trials with a reaction time <200 ms were rejected as outliers, and mean reaction times were only calculated for correct trials. To measure the ability to identify congruency, the correct response for an incongruent stimulus was regarded as a ‘hit’, whereas an incorrect response was regarded as a ‘miss’. An incorrect response for a congruent stimulus was regarded as a ‘false alarm’. The *d*′ values were calculated by transforming the response proportion to *z* scores and then subtracting the *z* score corresponding to the hit rate from the *z* score corresponding to the false-alarm rate (Stanislaw and Todorov, 1999). Repeated-measures analysis of variance (ANOVA) was conducted with the between-subject factor of group (expert and control) and the within-subject factor of condition (halfpipe trick and walking). The questionnaire scores were analyzed using the Mann–Whitney test to investigate intergroup differences.

#### EEG preprocessing and analysis.

EEG data were processed and analyzed using Letswave 7 (https://letswave.cn), which is an open-source MATLAB toolbox for neurophysiologic data analysis. EEG data were high-pass filtered with 0.1 Hz for event-related potential (ERP) analysis and low-pass filtered with 30 Hz (Butterworth filter, slope: 24 dB/oct). Additionally, a 50 Hz notch filter was used to remove noise. Ocular artifacts, and any other remaining artifacts, were isolated by independent component analysis algorithm decomposition and visual inspection. For ERP analysis, continuous EEG data were segmented from 200 ms before the onset of the follow-up stimulus to 1200 ms after onset. For the time–frequency (TF) analysis, data were segmented from 1500 ms before the onset of the video to 1000 ms after the onset of the follow-up stimulus (Figure 1C). All trials, except those with large artifacts (amplitudes exceeding ±100 mV), were included in the analyses. Over 98% of trials were retained for each condition. The statistical results of the number of remaining trials showed no significant difference between the different conditions/groups (all pairwise comparison *P*-values were >0.05).

**Fig. 3.**
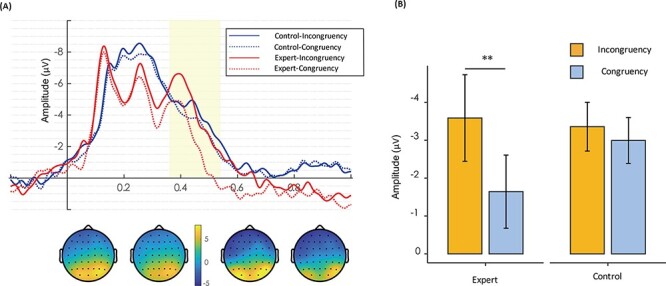
(A) Grand average N400 waveforms (360–540 ms) over bilateral frontal sites of experts and controls during the halfpipe trick. The solid lines represent the incongruent condition and the dotted lines represent the congruent condition. (B) Summary data for the mean amplitude values of the N400 component. Larger negativity was found in the incongruent condition compared with the congruent condition only in experts. Error bars represent the standard error of the mean. **P < 0.01 for the indicated comparison.

ERP data were baseline-corrected using data obtained from −200 ms to 0 ms. The mean amplitude in the time window from 360 ms to 540 ms was analyzed for the N400 component. Existing studies indicated the action-N400 is more frontal distributed (Reid and Striano, 2008; Proverbio and Riva, 2009; Amoruso *et al.*, 2013), with the grounded topographic map showing a more negative N400 electrophysiological activity in the frontal area (Figure 3A). Therefore, the area of interest consisted of six electrodes on the bilateral frontal area of the brain (left hemisphere: F3, F5 and AF3; right hemisphere: F4, F6 and AF4). Two repeated-measures ANOVAs were separately conducted with the between-subject factor of the group (expert and control) and the within-subject factor of congruency (congruent and incongruent) for the halfpipe trick and for walking.

The continuous wavelet transform was used for the TF decomposition of segmented EEG data for the frequency range between 1 and 30 Hz. A complex Morlet wavelet was selected as the mother wavelet, with a time decay parameter of 1.0 s and a center frequency of 1.5 Hz. Power values were normalized relative to the baseline period (−1250 to −250 ms before the video onset) and finally transformed into decibels by multiplying the log ratio by a factor of 10 (Grandchamp and Delorme, 2011).

**Fig. 4.**
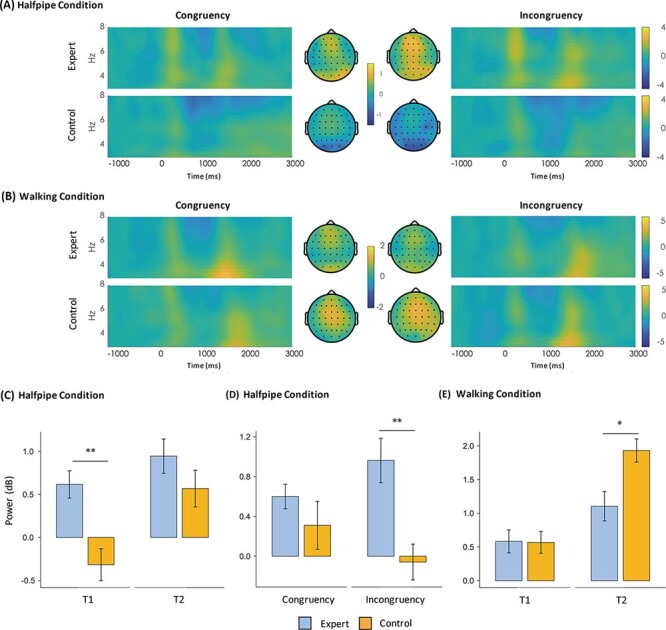
TF representations of the halfpipe (A) and walking (B) conditions represent the average of the midline sites (FCz, Cz and CPz). The topographic maps show the topographic distribution of theta activity from 500 to 1500 ms. Experts show greater theta oscillations than controls in T1 (C) and the incongruent condition (D) when observing halfpipe movements and show lower theta oscillations in the post-stimulus segment when observing walking movements (E). Error bars represent the standard error of the mean. *P < 0.05, **P < 0.01 for the indicated comparisons.

The frequency band was defined based on the individual alpha peak frequency (IAPF) that was defined as the maximum power density peak between 6 and 14 Hz. The IAPF was individually computed for each participant. The average IAPF was 10.430 Hz (±0.677, standard error, SE) in the expert group and 9.963 Hz (±1.108, SE) in the control group. There was no statistically significant difference between the two groups in the IAPF as assessed by a t-test (*P* = 0.440). Based on the IAPF, we estimated the theta band from IAF-6Hz to IAF-2Hz (Klimesch, 1999). On the basis of the previous study (Lu *et al.*, 2020) and the group averaged topography (Figure 4A,B), the fronto-centro-parietal midline brain area was selected as the region of interest (ROI), consisting of three electrodes: FCz, Cz and CPz. Two 500 ms segments were analyzed: before the stimulus onset (pre-stim time window, T1), 500–1000 ms, and after the stimulus onset (post-stim time window, T2), 1000–1500 ms. Repeated-measures ANOVAs were conducted with the between-subject factor of group (expert and control) and two within-subject factors, congruency (congruent and incongruent) and segment (T1 and T2), for the halfpipe trick and for walking.

#### Source reconstruction.

To estimate the sources of the ERPs and theta oscillation in the halfpipe judgment, standardized weighted low-resolution electromagnetic tomography (swLORETA) was used (Palmero-Soler *et al.*, 2007). The swLORETA method provides statistical parametric maps related to the reliability of the estimated density distribution of the current source. This method provides a discrete linear solution to the inverse EEG problem. swLORETA calculates the standardized current source density at each of 6239 voxels in the gray matter and the hippocampus with 5 mm spatial resolution. Individual anatomical imaging data were not available in this study; instead, a three-shell spherical head model registered to the Montreal Neurological Institute (MNI) 152 standardized brain map was used (Kötter *et al.*, 2001). swLORETA weights were derived from 61-channel EEG electrodes (electrooculograms and reference sites were excluded). EEG activity in the N400 and theta oscillation time window were exported to sLORETA software for each group (expert and control) and condition (congruent and incongruent) separately. t-Tests were carried out on the log-transformed data using sLORETA built-in randomization procedures (the number of possible permutations between the two sets of data was 5000) to correct for multiple comparisons. A significance level of 0.05 was employed. Results were represented in MNI coordinates with the corresponding Brodmann area.

## Results

### Behavior and questionnaire

**Fig. 2.**
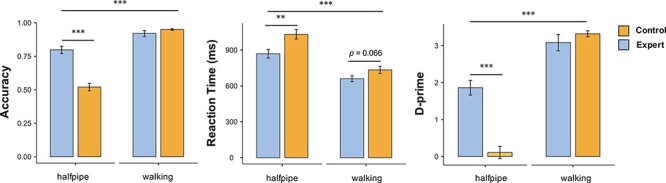
Behavioral performance in the task. There are significant differences in accuracy, reaction time and *d*′ scores between experts and controls in the halfpipe trick. Error bars represent the standard error of the mean. The upper-most comparisons are between the halfpipe condition and the walking condition overall, whereas the lower comparisons are between the experts and controls for each condition. **P < 0.01; ***P < 0.001.

The mean and standard deviation of each group’s behavioral performance are presented in Table 1. A comparison of accuracy showed significant main effects of group (expert *vs* control; *F*_1,28_ = 31.720; *P* < 0.001; $\rm{{\eta}_{p}^{2}}$ = 0.531) and of condition (halfpipe trick *vs* walking; *F*_1,28_ = 149.275; *P* < 0.001; $\rm{{\eta}_{p}^{2}}$ = 0.842). The two-way interaction between group and condition was also statistically significant (*F*_1,28_ = 46.162; *P* < 0.001; $\rm{{\eta}_{p}^{2}}$ = 0.622). The simple effects analysis of the interaction showed that the response accuracy of the experts was higher than that of the controls only in the halfpipe condition (*F*_1,28_ = 46.162; *P* < 0.001; $\rm{{\eta}_{p}^{2}}$ = 0.647). A comparison of reaction times showed significant main effects of group (*F*_1,28_ = 7.988; *P* = 0.009; $\rm{{\eta}_{p}^{2}}$ = 0.222) and of condition (*F*_1,28_ = 144.526; *P* < 0.001; $\rm{{\eta}_{p}^{2}}$ = 0.838). The two-way interaction between group and condition was also significant (*F*_1,28_ = 4.397; *P* = 0.045; $\rm{{\eta}_{p}^{2}}$ = 0.136). The simple effects analysis of the interaction showed that the experts responded faster than the controls in the halfpipe condition (*F*_1,28_ = 9.137; *P* = 0.005; $\rm{{\eta}_{p}^{2}}$ = 0.246) and in the walking condition (*F*_1,28_ = 3.648; *P* = 0.066; $\rm{{\eta}_{p}^{2}}$ = 0.115). The comparison of *d*′ values showed significant main effects of group (*F*_1,28_ = 20.228; *P* < 0.001; $\rm{{\eta}_{p}^{2}}$ = 0.419) and of condition (*F*_1,28_ = 147.123; *P* < 0.001; $\rm{{\eta}_{p}^{2}}$ = 0.840). The two-way interaction between group and condition was also significant (*F*_1,28_ = 29.709; *P* < 0.001; $\rm{{\eta}_{p}^{2}}$ = 0.515). The simple effects analysis of the interaction showed that *d*′ for experts was higher than that for controls only in the halfpipe condition (*F*_1,28_ = 45.279; *P* < 0.001; $\rm{{\eta}_{p}^{2}}$ = 0.618). Figure 2 illustrates the behavioral results. The Mann–Whitney analysis indicated significantly higher self-rating scores on action familiarity (*P* < 0.001), performance confidence (*P* = 0.001) and imagery engagement (*P* = 0.040) and lower scores on task difficulty (*P* = 0.007) among experts for the halfpipe condition and significantly higher scores on action familiarity (*P* = 0.031) and performance confidence (*P* = 0.022) among controls for the walking condition.

**Table 1.** Descriptive statistics of behavioral results

	Experts (*n *= 15)		Controls (*n *= 15)	
	Halfpipe	Walking	Halfpipe	Walking
	Mean ± s.d.	Mean ± s.d.	Mean ± s.d.	Mean ± s.d.
Reaction time (ms)	868.95 ± 140.45	659.80 ± 94.68	1031.36 ± 153.36	733.73 ± 116.22
Accuracy	0.80 ± 0.11	0.92 ± 0.08	0.52 ± 0.11	0.95 ± 0.02
*d*′	1.86 ± 0.77	3.08 ± 0.86	0.11 ± 0.65	3.32 ± 0.32

### N400

The analysis for the halfpipe trick revealed a significant main effect of congruency on the mean amplitude of the ERP component N400 (*F*_1,28_ = 17.096; *P* < 0.001; $\rm{{\eta}_{p}^{2}}$ = 0.379) and a significant two-way interaction between congruency and group (*F*_1,28_ = 8.007; *P* = 0.009; $\rm{{\eta}_{p}^{2}}$ = 0.222). The simple effects analysis of the interaction showed that the N400 mean amplitude for the incongruent condition was more negative than that of congruent condition only in the experts (*F*_1,28_ = 24.251; *P* < 0.001; $\rm{{\eta}_{p}^{2}}$ = 0.464). Figure 3 plots these values for illustrative purposes. In the walking condition, no statistically significant main effect or interaction was found.

### Theta oscillations

Theta oscillations were subjected to a 2 × 2 × 2 (group × congruency × segment) repeated-measures ANOVA. For the halfpipe condition, significant main effects of group (*F*_1,28_ = 4.680; *P* = 0.039; $\rm{{\eta}_{p}^{2}}$ = 0.143) and of segment were found (*F*_1,28_ = 22.396; *P* < 0.001; $\rm{{\eta}_{p}^{2}}$ = 0.444). A marginally significant two‐way interaction between congruency and group was detected (*F*_1,28_ = 4.142; *P* = 0.051; $\rm{{\eta}_{p}^{2}}$ = 0.129). The simple effects analysis of this interaction revealed that experts exhibited greater theta oscillations than controls only in the incongruent condition (*F*_1,28_ = 7.965; *P* = 0.009; $\rm{{\eta}_{p}^{2}}$ = 0.221). A significant two‐way interaction between segment and group was also observed (*F*_1,28_ = 4.685; *P* = 0.039; $\rm{{\eta}_{p}^{2}}$ = 0.143). The simple effects analysis of this interaction revealed that experts exhibited greater theta oscillations than controls only in T1 (*F*_1,28_ = 10.202; *P* = 0.003; $\rm{{\eta}_{p}^{2}}$ = 0.267).

For the walking condition, we detected a significant main effect of segment (*F*_1,28_ = 146.151; *P* < 0.001; $\rm{{\eta}_{p}^{2}}$ = 0.839) and a significant two‐way interaction between segment and group (*F*_1,28_ = 29.243; *P* < 0.001; $\rm{{\eta}_{p}^{2}}$ = 0.511). The simple effects analysis of this interaction indicated that experts exhibited lower theta oscillations than controls (*F*_1,28_ = 7.629; *P* = 0.010; $\rm{{\eta}_{p}^{2}}$ = 0.214). No main effect of condition or group was found. Figure 4 illustrates the findings for the N400 ERP component.

### Source reconstruction

**Fig. 5.**
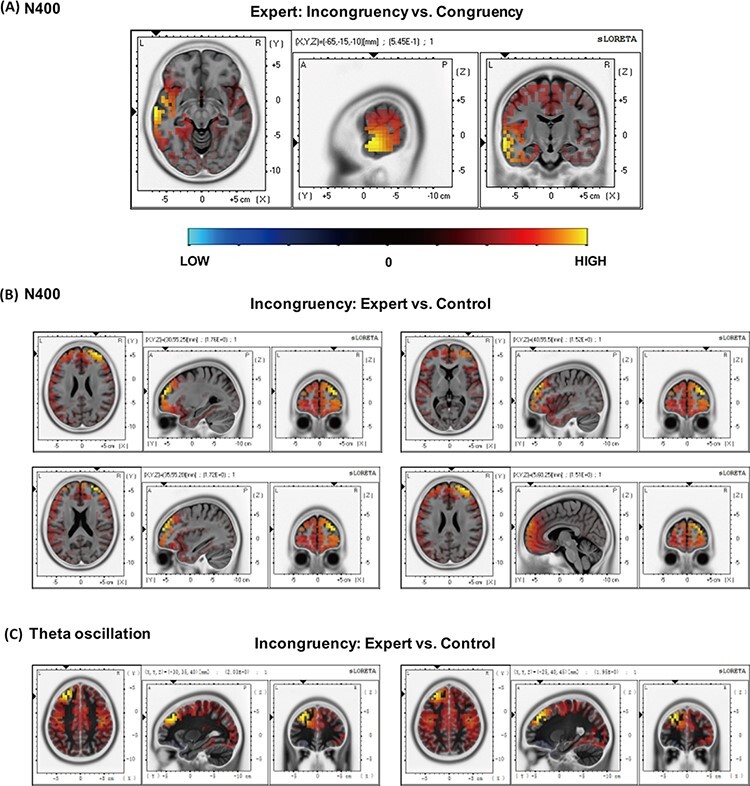
The main effects of congruency in experts and of expertise on the amplitude of the N400 and theta oscillation. Brain slices in each set of panels from left to right: axial (viewed from the top), sagittal (viewed from the left) and coronal (viewed from the back). L represents, left; R, right; A, anterior; and P, posterior. Coordinates in MNI space correspond to the maximum t statistic value, color-coded from (low) light-blue to (high) yellow. (A) Incongruency *vs* congruency in the expert group showing increased activation in the middle frontal gyrus. (B) Experts *vs* controls in the incongruent condition showing increased activation throughout several frontal areas, including the middle frontal gyrus, superior frontal gyrus, inferior frontal gyrus and medial frontal gyrus. (C) The theta oscillation source reconstruction: incongruency *vs* congruency in the expert group showing increased activation in the middle frontal gyrus and superior frontal gyrus.

The reconstruction of EEG activities in the halfpipe condition found that the localization of N400 in the expert group indicated a significantly increased activation in the middle temporal gyrus during the incongruent condition relative to the congruent condition. A comparison between the two groups for the incongruent condition indicated an increased activation for the experts in an extensive frontal area that included the middle frontal gyrus, superior frontal gyrus, inferior frontal gyrus and medial frontal gyrus. The localization of theta oscillations found that experts exhibited a higher activation of the middle frontal gyrus and superior frontal gyrus in the incongruent condition compared with the congruent condition in the T2 time window. The results of the source reconstruction are shown in Table 2 and Figure 5.

**Table 2.** Results of the source reconstruction in the halfpipe condition

			MNI coordinates			
Brain region	Hemisphere	Brodmann area	*x*	*y*	*z*	*t* Value
N400						
Incongruent: expert *vs* control						
Middle frontal gyrus	R (Right)	10	30	55	25	1.764
Superior frontal gyrus	R	10	35	55	20	1.720
Inferior frontal gyrus	R	10	40	55	5	1.516
Medial frontal gyrus	R	10	5	60	25	1.507
Expert: incongruent *vs* congruent						
Middle temporal gyrus	L (Left)	21	−65	−15	−10	0.545
Theta oscillation in T2 time window						
Expert: incongruent *vs* congruent						
Middle frontal gyrus	L	9	−30	35	40	2.029
Superior frontal gyrus	L	8	−25	40	45	1.954

## Discussion

This study aimed to examine whether congruency monitoring occurs at the motor level and how expertise influences this process. We compared the ability of expert snowboarders and controls to monitor and identify incongruencies in occluded video clips of a snowboarding halfpipe trick *vs* regular walking. These two activities represent whole-body actions that are not object- or goal-directed. We analyzed the electrophysiological activity in the brain of the participants to compare the activation in the experts and controls during action congruency monitoring.

In the action congruency judging task, experts performed better than controls in the snowboarding halfpipe trick condition. In contrast, both groups performed similarly when judging congruency in the walking condition. In addition, the incongruency of halfpipe trick only elicited greater N400 amplitude in experts but not in controls. These findings were in line with existing research that incongruency or error of action can be identified by experienced individuals and elicited N400 effect (Proverbio *et al.*, 2012; Amoruso *et al.*, 2014; Panasiti *et al.*, 2016). The N400 effect was previously detected during observations of unexpected-end actions (Amoruso *et al.*, 2013) or during semantic conflict processing (Proverbio and Riva, 2009). When observing actions, N400 might be associated with a neurocognitive mechanism involved in reconstructing the action meaning through previous experiences and current contextual information (Amoruso *et al.*, 2013). This effect also reflects the automatic recognition of minimal differences in the temporal and spatial dynamics of action (Orlandi *et al.*, 2017). In the present study, N400 amplitude was not modulated in controls when they were observing a domain-specific action. This result indicates the inability of controls to detect incongruency, due to their lack of knowledge about the corresponding motor repertoire. It is difficult for controls to create correct expectations of an observed action and match these expectations with their representations, especially when the goal-level information is lacking. Therefore, our finding that only skilled individuals can detect subtle incongruency when observing domain-specific action with no clear goal-level information supports our hypothesis. The present finding suggests the congruency monitoring can occur at the motor level and is enhanced by expertise.

In addition, both experts and controls showed a similar ability to detect incongruency in walking actions accurately. Although controls obtained higher scores in confidence and familiarity in the walking condition, no difference was found in their congruency judging performance and N400 activity. This difference in subjective ratings might be attributed to the comparison between halfpipe and walking. In contrast to the halfpipe condition, controls were, logically, more familiar with the walking actions and, hence, more confident of their performance. Furthermore, no N400 effect was found in both groups when judging walking actions. One explanation for this finding is that the congruency monitoring of simple daily actions without goals (i.e. walking) might not involve the meaning constructing process that N400 activity reflects in.

Furthermore, theta oscillation differed between experts and controls, especially for the halfpipe condition. Midline theta oscillations were higher in experts observing incongruent domain-specific actions in our analyses, supporting a previous study showing that theta activity is sensitive to the congruency between expected and observed actions and is modulated by an individual’s expertise (Lu *et al.*, 2020). Theta activity of the midfrontal brain areas during action monitoring is involved in conflict detection and the resolution process (Cavanagh *et al.*, 2012; van Driel *et al.*, 2015). Theta activity is also influenced by the functional demands of cognitive control (Cavanagh and Frank, 2014). In contrast, the control group could not form a valid expectation of the viewed halfpipe action, or monitor incongruency, due to the lack of an existing motor repertoire. Thus, these findings suggest that the expertise of given individuals can contribute to monitoring congruency at the motor level, involving in the conflicting processing and increasing cognitive control. In addition, experts also exhibited greater theta oscillations compared with controls before the follow-up stimulus in the halfpipe condition. The increasing midfrontal theta activity is not only elicited by errors and conflicts (Luu *et al.*, 2004; Debener, 2005) but also occurring before a target is presented, as an anticipatory and preparation of future conflict processing (van Driel *et al.*, 2015). The neural activity observed before a possible incongruency is presented might reflect the activation of the congruency monitoring process and preparation for response. These findings extend those of previous studies by demonstrating that the modulation generated by motor experience is not limited to the detection and identification process after incongruency occurs but also affects monitoring mechanisms that are activated in advance.

In the source reconstruction of theta oscillation, experts exhibited a greater frontal activation in the incongruent condition compared with the congruent condition. In addition, the localization of N400 in the present study indicated the extensive activation of the prefrontal area in experts compared with controls when identifying incongruent halfpipe actions. The prefrontal area has been implicated in some higher-level cognitive processes such as decision-making and executive functions that are associated with top–down processing (Miller and Cohen, 2001; Volz *et al.*, 2006; Yang and Raine, 2009). Thus, the monitoring and decision-making during action observation might be influenced by top–down modulation originating from the prefrontal area (Mansouri *et al.*, 2007, 2009). Frontal theta activity is also associated with the encoding and retrieval of memory in words and non-speech situations (Crespo-Garcia *et al.*, 2010; Klimesch *et al.*, 2010), especially when processing biological motion (Urgen *et al.*, 2013). Furthermore, the N400 localization of experts exhibited a greater activation of the middle temporal gyrus for the incongruent stimulus compared with the congruent one. The middle temporal region, which is part of the action processing pathway, is associated with representing the meaning of actions (Möttönen *et al.*, 2016). The activation of the middle temporal gyrus might reflect the processing of coding for the relationship between an observed action and its meaning based on an individual’s knowledge (Amoruso *et al.*, 2018; Papeo *et al.*, 2019). Abstract-level action understanding involves a ventral brain pathway linking the middle temporal gyrus to the inferior frontal gyrus (Kilner, 2011). The activation of the ventral pathway in experts suggests that expertise might help individuals reconstruct the meaning of observed actions, despite being presented at the motor level with no clear goal.

Furthermore, empirical studies and meta-analyses showed that long-term expertise training could change the neural plasticity of individuals, which was reflected in better processing speeds and specific neural activities (Nakata *et al.*, 2010; Voss *et al.*, 2010). Motor preparation and top–down control detected by electrophysiological activities were enhanced in athletes when compared with controls in previous studies(Bianco *et al.*, 2017a,b). In this study, better performance and greater frontal activity in experts, especially in the expertise-domain task, might reflect cognition superiority due to long-term training. During action observation, the intervention of top–down control facilitates the monitoring process and helps individuals identify subtle incongruences. In addition, the faster response (*P* = 0.066, considered marginally significant) and lower theta oscillation of experts in the walking condition might indicate the more efficient processing and lower cognitive demands when performing a common task.

In conclusion, the present study showed that congruency monitoring can occur at the motor level during action observations and is modulated by an individual’s expertise. Expertise for the observed action can generate a monitoring-related neural activity, which is reflected by theta oscillations rising after incongruency and before an ensuing possible incongruency. This phenomenon helps match the observed motor pattern with that expected based on the expert’s experience. The current study extends existing knowledge on action understanding, providing new mechanistic insights on congruency monitoring.

However, some limitations to this study should be noted. Theta oscillation and N400 activity were used to reflect action congruency monitoring and detection; however, a potential link between the two indexes was not elucidated. Future studies should explore how different brain activities during action congruency monitoring interact with each other or operate independently. In addition, even though the present study suggests expertise facilitates the congruency monitoring by the activation of the frontal and temporal brain regions, electrophysiological recordings cannot provide precise spatial evidence due to technical limitations. By utilizing neural imaging and stimulation intervention techniques, future studies can elucidate the neural pathway of congruency monitoring and how expertise influences the top–down modulation originating from frontal regions.
